# Virtual Herbarium ALTB: collection of vascular plants of the Altai Mountain Country

**DOI:** 10.3897/BDJ.9.e67616

**Published:** 2021-07-13

**Authors:** Aleksey V. Vaganov, Alexander I. Shmakov, Sergey V. Smirnov, Nadezda A. Usik, Alena A. Shibanova, Aleksey A. Kechaykin, Petr A. Kosachev, Tatyana M. Kopytina, Elizaveta A. Zholnerova, Kristina E. Medvedeva, Vladimir F. Zaikov, Tatyana A. Sinitsyna, Alexander P. Shalimov, Evgenij V. Antonyuk, Polina D. Gudkova, Denis A. Dmitriev, Alexander A. Batkin, Dmitry E. Kasatkin, Denis L. Belkin

**Affiliations:** 1 Altai State University, Barnaul, Russia Altai State University Barnaul Russia; 2 Sakhalin Branch of the Botanical Garden-Institute FEB RAS, Yuzhno-Sakhalinsk, Russia Sakhalin Branch of the Botanical Garden-Institute FEB RAS Yuzhno-Sakhalinsk Russia; 3 Tomsk University, Tomsk, Russia Tomsk University Tomsk Russia

**Keywords:** Altai Mountain Country, ALTB, collections, dataset, digital herbarium, Flora Altaica, plants occurrence, specimen

## Abstract

**Background:**

The herbarium of the South-Siberian Botanical Garden of Altai State University (ALTB) houses the largest collection of plants from the Altai Mountain Country (AMC), an area that extends across Russia, Kazakhstan, Mongolia and China. The collection of ALTB includes more than 450,00 specimens, making it the seventh largest in Russia and the fourth largest amongst Russian university herbaria. Altai State University (ASU), the home of ALTB, is one of the most important centres of academic education and research in Siberia and the Russian Far East. It is a sociocultural centre that provides a distinguished learning environment for undergraduate and graduate students in many scholarly and professional fields, meeting the needs of today's knowledge-based post-industrial society and contributing to regional development. It actively promotes international cooperation and strategic collaboration amongst countries of the AMC in the fields of science, education and culture. In particular, the activities of the South-Siberian Botanical Garden include: development of measures to protect rare and endangered plant species, research on the flora and vegetation of the AMC, preparation and publication of a multi-volume work "Flora Altaica", monographic study of individual plant groups, conducting laboratory classes, summer practicals and special courses. The main purpose of this article is to attract the attention of the scientific community to the botanical research of transboundary territory of the Altai Mountain Country (Russia, Kazakhstan, China and Mongolia) and to the future development of digital plant collections in partnership with Global Biodiversity Information Facility (GBIF).

**New information:**

The Virtual Herbarium ALTB (Russian interface - altb.asu.ru) is the largest digital collection of plants from the transboundary territory of the Altai Mountain Country and the main source of primary material for the "Flora Altaica" project (http://altaiflora.asu.ru/en/). Since 2017, when Altai State University became a GBIF data publisher, data from the Virtual Herbarium ALTB has been exported to the dataset "Virtual Herbarium ALTB (South-Siberian Botanical Garden)" in GBIF. Currently, it includes images and data from 22,466 vascular plants, of which 67% have geographic coordinates (accessed on 30.03.2021). Most of the specimens have been collected since 1977, with the most intensive collecting years being 1995–2008. In 2019, the label-data table of the Virtual Herbarium ALTB was modified to bring it into conformity with the Darwin Core specification (http://altb.asu.ru/). This effectively solved the major impediment to sharing plant diversity data from the AMC and adjacent regions in a multilingual environment.

## Introduction

The Altai Mountain Country (AMC) is the highest modern uplift amongst the continental mountain countries in Siberia, as well as in Northern and Central Asia in general (Kamelin 1998). This area occupies about 550,000 km^2^ including the Chinese, Kazakh, Mongolian and Russian Altai, as delimited by Kamelin (Flora Altaica, Kamelin and Shmakov 2005). More than 2,700 plant species, 300 of which are endemic, grow within the territory of the AMC. In 2002, David Olson and Eric Dinerstein singled Altai-Sayan territory as one of the 200 priority ecoregions of the world for global conservation of biodiversity in their work "The global 200 Priority ecoregions for global conservation" (Olson and Dinerstein 2002).

Due to the exceptional diversity of environmental conditions, the vegetation cover of the Altai Mountain Country is also highly diverse in its different parts and, in some of them, it is exceptionally diverse (Flora Altaica, Kamelin and Shmakov 2005). The principal features of the distribution of the vegetation are determined by its geographic position and its location at the junction of two different sectors of biodiversity in northern Asia, the West Siberian – Middle Asian – Himalayan and the Central Siberian – Central Asian – Indochinese sectors.

The herbarium of the South-Siberian Botanical Garden (SSBG) of Altai State University (ASU) was added to the Index Herbariorum database in 1996 under the code ALTB. ALTB has the largest collection of plants from transboundary territory of the Altai Mountain Country.The foundation of the primary collection of Herbarium ALTB was connected with the establishment of Altai State University in 1973 and of the South-Siberian Botanical Garden in 1979. In 1983, the University directive aimed to create a herbarium that included 5,000 sheets of plants from mountainous and plain parts of Altai Krai, collected by I.V. Vereschagina, A.I. Shmakov, T.A. Terekhina, E.P. Prokofjev, V.P. Kutafjev and G.G. Sokolova. N.V. Revjakina was responsible for primary systematisation, arrangement and addition to the collection (Usik 1999).

The herbarium's growth was stimulated in its early days by the extensive expeditions led by the SSBG and by the establishment of three scientific journals that focused on the regions flora: "Flora and vegetation of Altai" (editor A.I. Shmakov), "Turczaninowia" (editor R.V. Kamelin, from 2016 – A.I. Shmakov), "Problems of botany of South Siberia and Mongolia" (editor A.I. Shmakov) and "Botanical researches of Siberia and Kazakhstan" (editor A.N. Kuprijanov). In 2005, by the time the first volume of "Flora Altaica" was published, the Herbarium ALTB had more than 300,000 sheets. Since then, the collection has grown and now it contains more than 450,000 sheets. It ranks fourth in Russia amongst university collections and collections of institutes of the Academy of Sciences. Even today, numerous new specimens are collected yearly.

In 2009, the Virtual Herbarium ALTB was designed, based on the collections from ALTB and the experience of European herbaria. The registration of the corresponding database with digital images was made on 11 January 2010 (Federal Institute of Industrial Property, №2010620024). The creation of the virtual herbarium at that time was motivated by the need to protect the collections of the botanical garden, which is particularly important for type materials (so the sheets and the information on them can be accessed without damage) (Vaganov 2011, Vaganov and Medvedeva 2020). Nowadays, however, digitisation of collections is required for the world’s biodiversity research. It is a powerful technique that enables sharing information and increasing management efficiency by providing quick access to the specimens and their labels. Public access to such information via the Internet makes collections more broadly useful and improves scientific research on the originals (Smith and Blagoderov 2012, Seregin 2018). In addition, it reduces the time and money curators and researchers used to spend in correspondence and shipping specimens between institutions (Shashkov et al. 2018).

The "Flora Altaica" project (altaiflora.asu.ru) was initiated in 2018. It is designed to foster collaboration amongst botanists working on the flora of the AMC. The draft sections include, amongst other things, original layouts of the printed version of multi-volume "Flora Altaica", the keys of taxa and the AMC map. The map has 19 botanical-geographical areas (polygons) in the GeoJSON specification. The use of GeoJSON polygons, as well as the Shapefile obtained on their basis, makes it easy to generate distribution maps using GIS-programmes, thereby clarifying the current range of the taxa involved and facilitating the task of citing representative specimens. The AMC polygons are available for downloading to all interested users in the section "AMC Map" of the project.

The biodiversity of the AMC territory has been explored for over 200 years. The first report on the flora of Altai was written by C.F. von Ledebour and his disciples C.A. von Meyer and A.G. von Bunge and published in 1829 in four volumes (Ledebour C. F. 1829). The next edition on the flora of Altai under the title "Flora of Altai and Tomsk Province" was completed by P.N. Krylov at the turn of the 20th century. In the modern period, a critical summary "Flora of Western Siberia" touched upon the Russian part of the AMC territory.

Only the herbarium samples can reliably confirm the presence of the plant organism in a specific point of space at a certain time. Herbarium collections and the data they hold are valuable, not only for the traditional studies of taxonomy and systematics, but also for ecology, bioengineering, conservation, food security and the human social and cultural elements of scientific collection (Baird 2010, James et al. 2018, Kovtonyuk et al. 2020).

## General description

### Purpose

The main purpose of this article is to attract the attention of the scientific community to the botanical research of transboundary territory of the Altai Mountain Country (Russia, Kazakhstan, China and Mongolia) and to the future development of digital plants collections in partnership with Global Biodiversity Information Facility (GBIF).

## Project description

### Title

Scientific depository of phytodiversity of Altai Krai and the adjacent territory of the Altai Mountain Country (Russian Foundation for Basic Research project №19-44-220004).

### Study area description

The exact boundaries of the AMC were presented in 2005 by 19 botanical and geographical areas in the first volume "Flora Altaica". This zoning became the background for polygons edited in the GeoJSON specification for working in GBIF and converted to shapefiles for GIS programmes (data are available to all researchers at http://altaiflora.asu.ru/ru/карта-агс/).

The result of the first period grant implementation to Global Biodiversity Information Facility (GBIF) from the ALTB Foundation (Altai State University) includes more than 22,466 records, backed up by a digital image of the Herbarium. During the inventory work, original tables for the following plant groups were obtained: rare and endemic, invasive, economically valuable and other promising plant species. The team identified 1,176 medicinal plants and 296 food plants in the studied area. In the group "other promising plant species", we made significant inroads on digitising the material of large genera of such families as Caryophyllaceae, Rosaceae, Fabaceae, Scrophulariaceae and others. To add value to the data on the labels, the records were georeferenced. This increased the proportion of specimens with coordinates from 53% to 67% (Vaganov et al. 2021b).

### Design description

Development of Virtual Herbarium ALTB is an integral part of the larger "Flora Altaica" project. Its goals are to aid that project by:

1. Inventory of herbarium material ALTB for digitisation and preparation of primary data from labels on priority groups of plants: rare and endemic, invasive, economically valuable and other promising (monographic treatments of taxa).
2. Development of the Virtual Herbarium ALTB and maintenance of the digital collection on the national server of Altai State University.
3. Developing scientific skills in research teams on the Darwin Core specification, adjusting researchers' activities to the global standards of modern biology.
4. Regular publication of data in GBIF from the publisher "Altai State University".
5. Storage and processing of scientific data on phytodiversity of Altai Krai and adjacent territory of the AMC: establishment of new occurrences of studied objects, identification of plants habitats, modelling of habitat dynamics, based on possible locations of rare, economically valuable and invasive species and possibly new species for science.
6. Increasing the level of biodiversity knowledge about the AMC as one of the world's key ecoregions.

## Sampling methods

### Step description

The stages of herbarium digitisation are clearly described in many works (Seregin and Stepanova 2020, Svetasheva and Seregin 2020), one of the fundamental ones being Nelson et al. (2012). For the digitising of Herbarium ALTB, we implemented the following steps: pre-digitisation curation and staging, specimen image capture, specimen image processing, electronic label data capture, georeferencing specimen data.

**1. Pre-digitisation curation and staging**

The Curator of Herbarium ALTB selects for digitisation only mounted specimens with label, checked by plant taxonomists. Prior to digitisation, a barcode with the name of the herbarium "Herbarium ALTB" and a ten-digit number (for example, 1100000005) is glued to the herbarium sheet. The first three digits indicate to which major group the specimen belongs, "101" means ferns, horsetails and club mosses, "110" means seed plants. Other sequences have been set aside for other groups present in the collection, for example, 102 – lichens, 103 – mosses 104 – algae, 105 – fungi.

**2. Specimen image capture**

From 2007 to 2012, type material and representatives of the family Caryophyllaceae were completely digitised (3,717 sheets) using the modified scanner (useful model patent "Herbarium sheet feeder", № 146036). Each sheet was placed on a soft foam mat, which also helped to reduce pressure on the sheet in the Mustek PageExpress A3 USB 600 scanner. Digital copies of the herbarium sheet were provided with the 12 basic palette colours standard (HSB model). The images were saved in JPEG format with a resolution of 2793 x 3969 pixels (primary DPI 150 – using camera, DPI 72 – on server). After scanning, each image was renamed according to its barcode, which serves as a unique identifier. After completion of the first phase, digitisation was more fragmentary, precedence being given to those relating to active research projects, including thesis projects.

In December 2017, Altai State University became a GBIF data publisher (www.gbif.org/publisher/943a5811-d56e-4c37-853d-bd64957d3833) and started a new phase of herbarium digitisation. In particular, images were recorded using a Canon-EOS 400D camera placed on a tripod. By the end of 2020, 22,466 images had been added to the ALTB database. In the near future, the imaging equipment will be changed again, to a Microtek 1600 Object Scanner. They will be recorded as TIFF files with a 600 dpi resolution and include a 24-sample colour standard.

**3. Specimen image processing**

The image is processed by cropping the margins that protrude beyond the borders of the herbarium sheet, manually renaming according to the voucher number and then saving it in *jpg format. Then prepared images are copied via SFTP to the server of Altai State University (www.asu.ru).

**4. Electronic data capture**

Information from the label was manually entered into the digital form which contained 17 fields: unique barcode number (occurrenceID); status of herbarium material (typeStatus); country of herbarium collection (country); taxonomic category of material (phylum); family name (family); genus name (genus); species name with author citation (acceptedNameUsage); internal number of the label (catalogNumber); collectors (recordedBy); exact place of collection (verbatimLocality); ecology (locationRemarks); coordinates (decimalLatitude, decimalLongitude); altitude above sea level (verbatimElevation); date of collection (eventDate); who identified the herbarium material (IdentifiedBy); notes (occurrenceRemarks); image (available / not available). The exact species name with its author citation was rechecked according the International Plant Names Index (IPNI). To manage the tables of the Virtual Herbarium ALTB database, we developed the same-named site (http://old.ssbg.asu.ru/altb_herbarium.php) in the PHP programming language using the web-interface MySQL phpMyAdmin 5.

In 2019, the table of accumulated label data of Virtual Herbarium ALTB was adapted to the Darwin Core specification (altb.asu.ru). Following this data standard resolved many technical problems encountered when attempting to share data about the phytodiversity of Altai Krai across countries and languages. The updated Virtual Herbarium ALTB website has been prepared using PHP programming language and Smarty template engine. The site interacts with the database and performs a structured visualisation of the information using the standardised HTML document markup language. In contrast to the previous version, the project used Yandex.Maps API to visualise the geographical location of the records, which allows the use of cartographic data and Yandex technologies in the project. The logic of the designed application allows an individual label to be formed of each herbarium sheet included in the Virtual Herbarium ALTB database with the ability to geo-position and zoom the image when hovering the cursor without the need to download the image (e.g. http://altb.asu.ru/page.php?page=1100035931).

**Figure 1.**
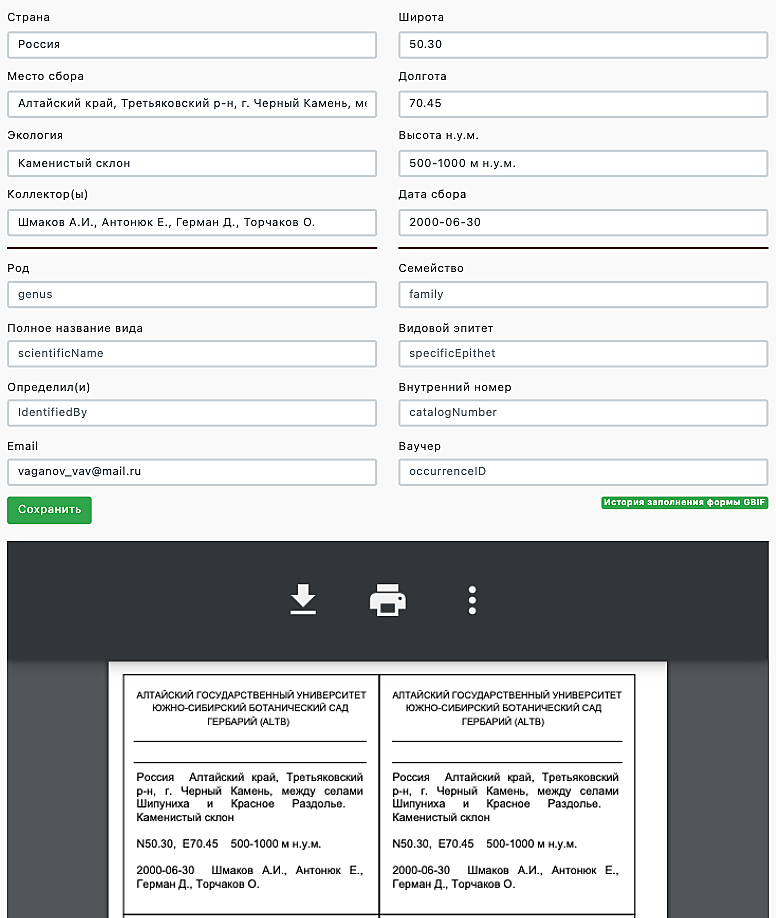
Interface of the form for filling the label information from the operator's personal office of the database "Virtual Herbarium ALTB" (altb.asu.ru, accessed on 30.03.2021).

In the current website version, label information is entered from the herbarium sheet directly into a designed data entry form in the personal office of the database operator. A printed label in PDF format is generated automatically when entering a label data and saving forms (Fig. 1). Forms saving by several operators continuously develop the single table that is available for download in csv-format. A table in csv-format has a final view in the Darwin Core specification for uploading to the Virtual Herbarium ALTB database and to GBIF through the IPT node (http://altb.asu.ru/ipt/). Verification of nomenclature names of the entire table of accumulated information ALTB since 2020 is additionally being performed in Global Names Resolver (http://resolver.globalnames.org) and Taxonomic name resolution service v.5.0 (https://tnrs.biendata.org).

**5. Georeferencing specimen data**

The Herbarium ALTB is relatively young, so the proportion of herbarium labels with coordinates was about 50%. Additional manual georeferencing keeps this share around 70%. Georeferencing accuracy of 500 m is carried out using standard electronic mapping libraries (Yandex.Maps, Google Maps).

## Geographic coverage

### Description

**Figure 2.**
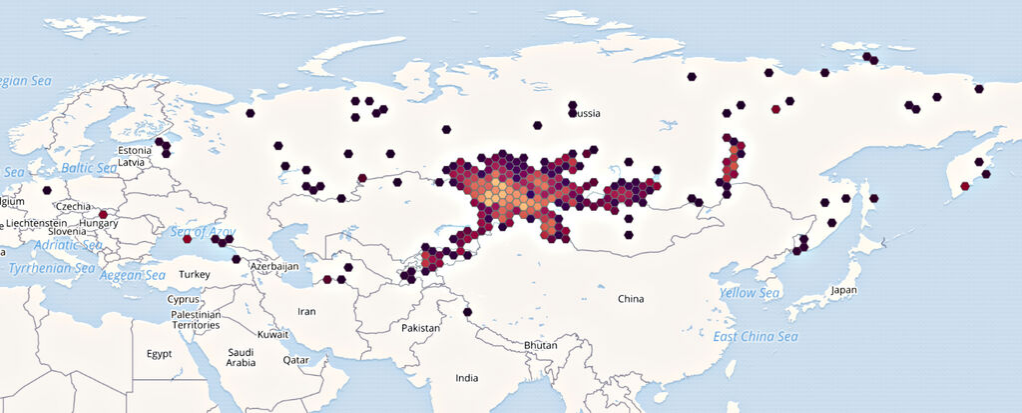
Geographic coverage of occurrences with precise coordinates digital collection ALTB (Virtual Herbarium ALTB (South-Siberian Botanical Garden) (GBIF.org, accessed on 30.03.2021).

The beginning of Herbarium ALTB digitising and global positioning of the available samples in GBIF showed the expected focus of the collections in the Altai Mountain Country (Fig. 2). The main expedition routes of the Herbarium’s donors lie within the south of Western Siberia of Russia, Eastern Kazakhstan, Western Mongolia and Northern China. A notable array of data is associated with the foothill part of the Altai Mountains – the south of Altai Krai and the Republic of Altai, the main places of collections made by the creators of the primary University Herbarium. At the same time, fragmentary, but still significant, collections from Central Asia (Kyrgyzstan, Tajikistan), Europe, the European part of Russia, Baikal and Transbaikal Siberia and the Far East add value to the collection. Type material from distant places is also present, primarily due to the development of the journal "Turczaninowia" and its section "new taxa".

### Coordinates

37N and 60N Latitude; 65E and 114E Longitude.

## Taxonomic coverage

### Description

**Figure 3.**
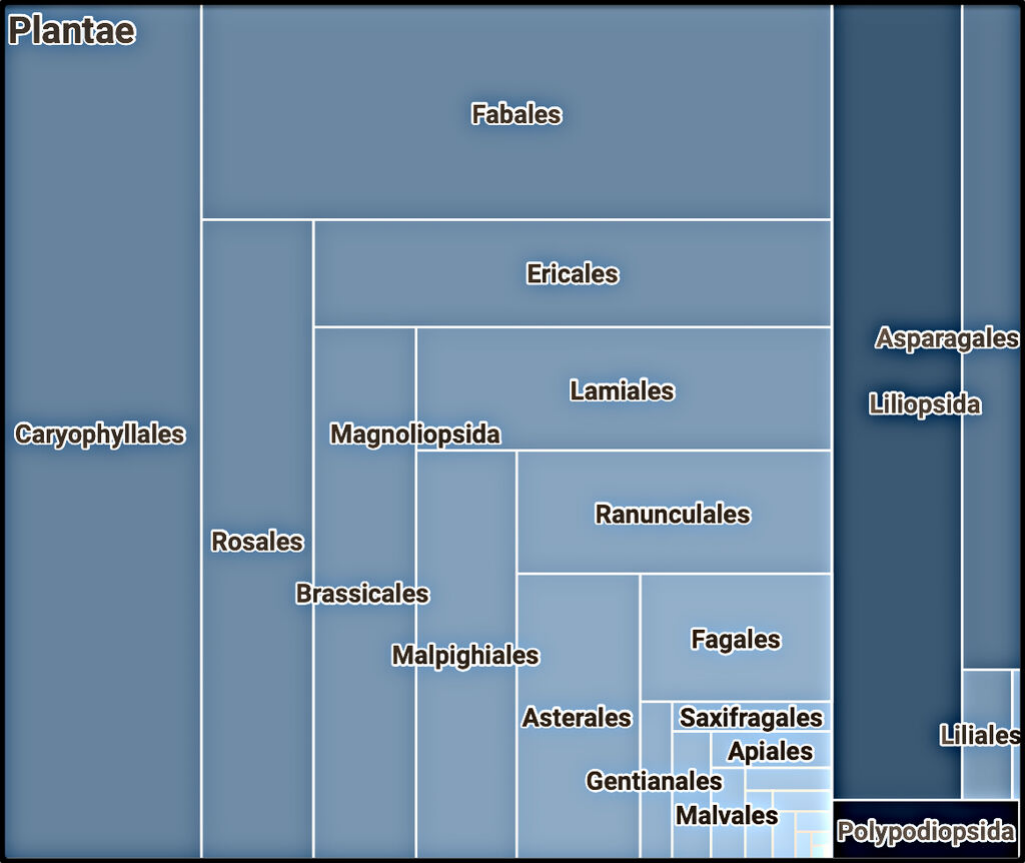
Taxonomic distribution of occurrences digital collection ALTB (Virtual Herbarium ALTB (South-Siberian Botanical Garden) (GBIF.org, accessed on 30.03.2021).

A taxonomic analysis of 22,466 occurrences from ALTB in GBIF showed the following taxonomic distribution of the records: ferns (Polypodiopsida) – 288 records, monocotyledons (Liliopsida) – 3,848 and dicotyledons (Magnoliopsida) – 18,330 (Fig. 3) (accessed on 30.03.2021). The sampling from the collection is determined by the purposes of the projects: "Flora Altaica" and "Scientific depository of phytodiversity of Altai Krai and the adjacent territory of the Altai Mountain Country". For the "Flora Altaica" project, the type material was digitised first. Currently, the focus is on digitising the following taxonomic groups: Rosaceae, Fabaceae, Scrophulariaceae, Brassicaceae, Alliaceae and Poaceae. The choice is dictated by the presence of plant taxonomists interested in these groups, who process and study taxa within the framework of projects and are ready to enter into communication in case of mutual interest with other specialists. Within the project "Scientific depository of phytodiversity of Altai Krai and the adjacent territory of the Altai Mountain Country", the following groups of plants were identified for digitisation: rare plants of the Altai Krai (the Red Data Book) and currently rare and endemic species of the AMC (Vaganov et al. 2021a); families that include or with representatives having obvious economic value (Aristolochiaceae, Berberidaceae, Paeoniaceae, Papaveraceae, Hypecoaceae, Fumariaceae, Portulacaceae, Limoniaceae, Betulaceae, Hypericaceae, Juglandaceae, Ericaceae, Vacciniaceae, Pyrolaceae, Empetraceae, Primulaceae) and invasive taxa (Ovcharova and Vaganov 2020).

## Usage licence

### Usage licence

Other

### IP rights notes

This work is licensed under a Creative Commons Attribution (CC-BY) 4.0 Licence (http://creativecommons.org/licenses/by/4.0/). The licence covers images of the herbarium specimens deposited in http://altb.asu.ru and available in GBIF, as well as their metadata.

## Data resources

### Data package title

Virtual Herbarium ALTB (South-Siberian Botanical Garden)

### Resource link

https://doi.org/10.15468/y6xmme

### Alternative identifiers

https://www.gbif.org/dataset/2dda31ff-f776-452f-9a4b-d5229f6e3494, http://altb.asu.ru/index.php

### Number of data sets

1

### Data set 1.

#### Data set name

Virtual Herbarium ALTB (South-Siberian Botanical Garden)

#### Data format

Darwin Core

#### Number of columns

22

#### Download URL

http://altb.asu.ru/ipt/resource?r=altb

#### Data format version

1.27

#### 

**Data set 1.** 

Column label	Column description
occurrenceID	An identifier for the Occurrence (as opposed to a particular digital record of the occurrence). In the absence of a persistent global unique identifier, construct one from a combination of identifiers in the record that will most closely make the occurrenceID globally unique. http://rs.tdwg.org/dwc/terms/occurrenceID
references	A related resource that is referenced, cited or otherwise pointed to by the described resource. http://purl.org/dc/terms/references
basisOfRecord	The specific nature of the data record. Included value: HumanObservation. http://rs.tdwg.org/dwc/terms/basisOfRecord
country	The name of the country or major administrative unit in which the Location occurs. http://rs.tdwg.org/dwc/terms/country
countryCode	The standard code for the country in which the Location occurs. http://rs.tdwg.org/dwc/terms/countryCode
family	The full scientific name of the family in which the taxon is classified. http://rs.tdwg.org/dwc/terms/family
genus	The full scientific name of the genus in which the taxon is classified. http://rs.tdwg.org/dwc/terms/genus
specificEpithet	The name of the first or species epithet of the scientificName. http://dwc/terms/specificEpithet
scientificName	The full scientific name. http://rs.tdwg.org/dwc/terms/scientificName
catalogNumber	An identifier (preferably unique) for the record within the data set or collection. http://rs.tdwg.org/dwc/terms/catalogNumber
recordedBy	A list (concatenated and separated) of names of people, groups or organisations responsible for recording the original Occurrence. http://rs.tdwg.org/dwc/terms/recordedBy
verbatimLocality	The original textual description of the place. http://rs.tdwg.org/dwc/terms/verbatimLocality
decimalLatitude	The geographic latitude (in decimal degrees, using the spatial reference system given in geodeticDatum) of the geographic centre of a Location. http://rs.tdwg.org/dwc/terms/decimalLatitude
decimalLongitude	The geographic longitude (in decimal degrees, using the spatial reference system given in geodeticDatum) of the geographic centre of a Location. http://rs.tdwg.org/ dwc/terms/decimalLongitude
minimumElevationInMetres	The original description of the elevation (altitude, usually above sea level) of the Location. http://rs.tdwg.org/dwc/terms/verbatimElevation
eventDate	The date-time or interval during which an Event occurred. For occurrences, this is the date-time when the event was recorded. Not suitable for a time in a geological context. http://rs.tdwg.org/dwc/terms/eventDate
IdentifiedBy	A list (concatenated and separated) of names of people, groups or organisations who assigned the Taxon to the subject. http://rs.tdwg.org/dwc/terms/identifiedBy
typeStatus	A list (concatenated and separated) of nomenclatural types (type status, typified scientific name, publication) applied to the subject. http://rs.tdwg.org/dwc/terms/typeStatus
locationRemarks	Comments or notes about the Location. http://rs.tdwg.org/dwc/terms/locationRemarks
associatedMedia	A list (concatenated and separated) of identifiers (publication, global unique identifier, URI) of media associated with the Occurrence. http://rs.tdwg.org/dwc/terms/associatedMedia
CoordinateUncertaintyInMetres	The horizontal distance (in metres) from the given decimalLatitude and decimalLongitude describing the smallest circle containing the whole of the Location. Leave the value empty if the uncertainty is unknown, cannot be estimated or is not applicable (because there are no coordinates). Zero is not a valid value for this term. http://rs.tdwg.org/dwc/terms/coordinateUncertaintyInMeters
language	A language of the resource. http://purl.org/dc/elements/1.1/language
